# Comprehensive Evaluation of Cryptic *Juglans* Genotypes: Insight from Molecular Markers and Phylogenetic Analysis

**DOI:** 10.3390/genes15111417

**Published:** 2024-10-31

**Authors:** Sajjad Sajjad, Muhammad Islam, Khushi Muhammad, Sajid-ul Ghafoor, Irfan Ullah, Asif Khan, Muhammad Siraj, Abdulwahed Fahad Alrefaei, Jawad Ali Shah, Sajid Ali

**Affiliations:** 1 Department of Biotechnology and Genetic Engineering, Hazara University Mansehra, Mansehra 21300, Pakistan; sajjad.pk43@gmail.com (S.S.); khushisbs@yahoo.com (K.M.); sajid@hu.edu.pk (S.-u.G.); irfan.qasami@gmail.com (I.U.); 2 Department of Technology, State University of Maringá, Umuarama 87506-370, PR, Brazil; asif.khan@alumni.usp.br; 3 Department of Biotechnology, Jeonbuk National University, Iksan 54596, Republic of Korea; sirajuom2@gmail.com; 4 Department of Zoology, College of Science, King Saud University, P.O. Box 2455, Riyadh 2455, Saudi Arabia; 5 Key Laboratory of Plant Reproductive Adaptation and Evolutionary Ecology and Institute of Biodiversity, Yunnan University, Ministry of Education, Kunming 650500, China; ali_jawad009@yahoo.com; 6 Department of Horticulture and Life Science, Yeungnam University, Gyeongsan 38541, Republic of Korea

**Keywords:** walnuts, DNA barcodes, GenBank, *ITS2*, *UBE3*, identification

## Abstract

**Background/Objectives:** The current research work aimed to evaluate the cryptic walnut genotypes of the Hazara region in Pakistan by using DNA barcoding and phylogenetic analysis. **Methods:** Based on morphological traits such as nut size, nut shape, and the number of leaflets, five genotypes were chosen and samples were collected for the current study. For molecular analysis, gDNA was isolated from the fresh leaves, and the five most effective angiosperm-specific markers, ITS2, *rbcLa*, *rbcLc*, *rpoC1*, and *UBE3*, were utilized. Based on amplification, sequencing, and identification success rates, ITS2 and *UBE3* were recorded as the most efficient markers followed by *rbcLa*, *rbcLc,* and *rpoC1*. **Results:** During phylogenetic analysis, the query genotype-1 based on ITS2 and genotype-2 based on *UBE3* clustered with (KF454101.1-*Juglans regia*) and (KC870919.1-*J. regia*) with bootstraps of 56 and 100, respectively. Genotype-3 based on *rbcla* clustered in a major clade with *J. regia* L., cultivars (MN397935.1 *J. regia* ‘Vina’) and (MN397934.1-*J. regia* ‘Serr’), (MN397933.1 *J. regia* ‘Pedro’), (MN397932.1 *J. regia* ‘Lara’), (MN397931.1 *J. regia* ‘Howard’), and (MN397930.1 *J. regia* ‘Hartley’) with bootstrap of 100. Meanwhile, genotype-4 and genotype-5 based on *rbclc* and *rpoC1* clustered with (MN397935.1 *J. regia* ‘Vina’) and (MN397934.1 *J. regia* ‘Serr’), across the database sequences. To clarify the taxonomic status of cryptic walnut genotypes, it is necessary to combine diverse DNA barcodes. The results of ITS2 and *UBE3*, followed by *rbcL* barcoding markers, are promising taxonomic tools for cryptic walnut genotypes in Pakistan. **Conclusions:** It has been determined that the genotypes of walnuts in the study area are both *J. regia* L. and its cultivars and that the accuracy of discrimination regarding the genus *Juglans* L. is greater than 90%. The reported DNA barcodes are recommended for the correct identification and genetic evaluation of *Juglans* taxa and its population.

## 1. Introduction

The genus *Juglans* L. (Juglandaceae), having basic chromosome number 2n = 32, is a monoecious in nature, wind-pollinated, and self-compatible flowering plant [1]. The Juglandaceae family comprises 10 genera and 60 species [2]. The *Juglans* L. genus is one of the major genera of the family Juglandaceae, consisting of 21 species [3]. The walnut holds significant economic and commercial importance due to its edible nuts, high-quality wood, suitability for landscaping and ornamental purposes, and traditional, cultural, and medicinal uses [4]. Recent epidemiological studies have linked walnut consumption to a reduced risk of cardiovascular mortality [5]. Walnuts originate from the mountainous areas of Central Asia and are found in a wide territory that extends from southeastern Europe and the Caucasus to Turkey and Iran [6]. They also grow in Armenia, Azerbaijan, Belarus, Estonia, Georgia, Kazakhstan, Kyrgyzstan, Latvia, Lithuania, Moldova, Russia, Tajikistan, Turkmenistan, Ukraine, and Uzbekistan, as well as in China and the eastern Himalayas [7].

The genus *Juglans* L. is classified into four major sections, *Cardiocaryon*, *Dioscaryon*, *Rhysocaryon,* and *Trachycaryon* [8]. *Cardiocaryon* consists of *Juglans ailantifolia* Carriere., *Juglans cathayensis* Dode, and *Juglans mandshurica* Maxim. *Dioscaryon* and *Trachycaryon* consist of single species *Juglans cinerea* L. and *J. regia* L., respectively. *Rhysocaryon* is endemic to South and North America and comprises *Juglans australis* Griseb., *Juglans boliviana* Dode, *Juglans californica* S., *Juglans guatemalensis* W.E. Manning, *Juglans hindsii* Jeps, *Juglans hirsuta* W.E. Manning, *Juglans jamaicensis C. DC.*, *Juglans major* Torr, *Juglans microcarpa* Berland, *Juglans mollis* Engelm, *Juglans neotropica* Diels, *Juglans nigra* L., *Juglans olanchana* Standl, *Juglans pyriformis* Liebm, *Juglans steyermarkii* W.E. Manning, and *Juglans venezuelensis* W.E. Manning [9]. All these species exhibit variation in nut size and shape, leaf shape, leaflet number, and overall morphological characteristics [10]. 

The walnut has a long cultivation history spanning over 6800 years, with more than 300 *J. regia* L. cultivars documented to produce edible nuts [11]. In Pakistan, walnuts predominantly grow in the northern region, with Hazara being the major contributor to walnut production and exhibiting rich morphological walnut diversity [12]. Despite the presence of numerous walnut cultivars and diversity in both wild and cultivated forms, their taxonomic status remains suspicious due to a lack of skilled experts, literature, comprehensive data, and accessibility to their habitat [13]. Remarkably, the flora of Pakistan reported only one species, *J. regia* L., approximately 70 years ago [14].

DNA barcoding is an emerging technological identification tool that performs identification of the genus level, species level, or even lower taxonomic levels of biological taxa [15]. The primary purpose of this technique is the correct identification of multiple plant species based on DNA fragment nucleotide sequences within a short duration [16]. It is an efficient, rapid, cost-effective, and standardized method for assessing and identifying various plant species [17]. Moreover, DNA barcoding allows for the exploration of both inter-specific and intra-specific variations and facilitates the identification of unknown species or those exhibiting complex morphometric behaviors [16]. Initially, COI gene sequences were used for the identification of certain metazoan species [18]; subsequently, various nucleotide sequences such as ITS, *matK*, *rbcL,* and *trn*H-*psb*A were employed for the identification of flowering plant species [19]. These nucleotide sequences play a crucial role in studying the similarities and differences among different plant species and are widely employed in addressing evolutionary and taxonomic issues [20]. Several plastid genome loci including *rbcL*, *matK rpoB*, *rpoC*1, and *trn*H-*psb*A have been explored and used as DNA barcoding regions for plants [21]. 

The current study was designed to evaluate the suspicious walnut genotypes found in the Hazara region of Northern Pakistan through molecular markers for their differentiation and identification and establish phylogenetic or evolutionary relationships with database reference sequences.

## 2. Materials and Methods

### 2.1. Study Area and Sample Collections 

**Figure 1:**
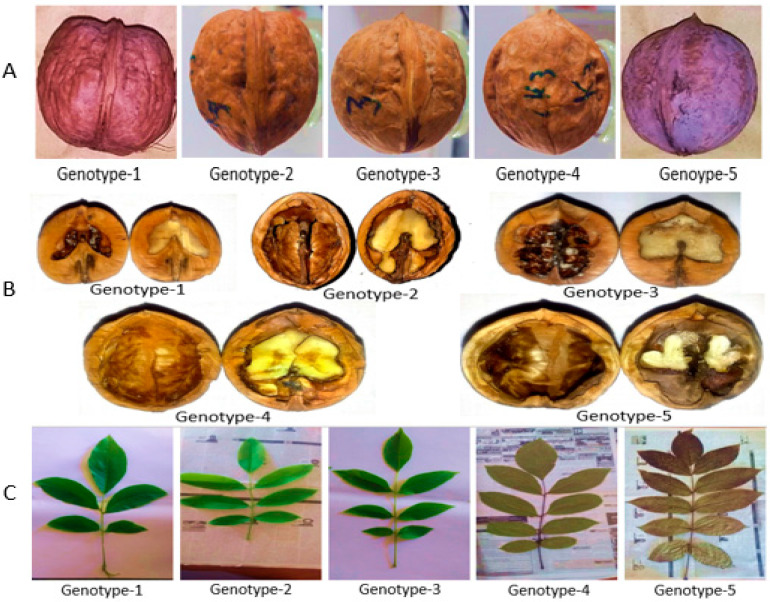
Nuts, nut cross-sections, and leaflet variations of walnut genotypes studied. (**A**) Nut shape, (**B**) nut cross-section, and (**C**) leaflets.

The current research work was conducted in the Hazara region of Khyber Pakhtunkhwa Province (KP), Pakistan. Initially, we collected 150 samples in the form of nuts and leaves. Further, these samples were segregated into fifteen groups based on their morphological traits. Again, two genotypes were randomly selected for the DNA barcoding of *Juglans* (Figure 1). After amplification and nucleotide sequencing, only five walnut genotypes were found suitable for further analysis. The samples were carefully tagged and stored at −20 °C in the laboratory (Table 1).

### 2.2. DNA Extraction and Quantification

For DNA isolation, the fresh leaf samples were powdered well using a mortar and pestle. About 100 mg of powder tissue of each genotype was added to an Eppendorf tube, 800 µL of the CTAB solution was added, and the mixture was vortexed thoroughly. Then the homogenate was incubated for 2 h at 65 °C. Following incubation, 600 µL of phenol-chloroform isoamyle alcohol (PCI) was added, and the homogenate was centrifuged for 20 min at 13,000 rpm. The supernatant, along with 500 µL of ice-cold isopropanol, was frozen for 2 h. Subsequently, the samples were centrifuged again at 13,000 rpm for 20 min, and the isopropanol was discarded. Next, 500 µL of ethanol (70%) was added, and the samples were centrifuged for 10 min at 13,000 rpm. The tubes were dried to remove the ethanol completely. To dilute the dried sample, 60–80 µL of ddH_2_O was added. The quality and concentration of the extracted gDNA were evaluated using a 1% agarose gel through electrophoresis [22,23,24].

### 2.3. Primers Selection and PCR Amplification

The internal transcribed spacer region (ITS2) [25], *UBE3* [26], and chloroplasts *rbcLa* [27], *rbcLc* [28], and *rpoC1;* CBOL [29] are the most studied and widely used nuclear DNA markers and were selected for molecular analysis of the suspicious walnut genotypes; the nucleotide sequences of their respective primers are presented in Table 2. In the amplification of the desired DNA fragment, a PCR reaction mixture was prepared in PCR tubes with a total volume of 25 µL for each DNA marker [30].

Each reaction mixture contained 14 µL of ddH20, 2 µL of template DNA, 2.5 µL of 10× PCR buffer, 2 µL of MgCl_2_, 2 µL of dNTPs, 0.5 units of Taq Polymerase kit (Catalogue no. KO171), and 2 µL of each forward and reverse primers. The amplification of the desired DNA markers was conducted using an applied Biosystems 2720 thermal cycler (PCR) (GRI, Braintree, United Kingdom) [31]. The conditions of PCR consisted of initial denaturation at 94 °C for 5 min, followed by 35 cycles of denaturation at 94 °C for 30 s, annealing for 40 s at 52 °C, extension at 72 °C for approximately 35 s, and a final extension step for 10 min at 72 °C [32]. The PCR-amplified products were analyzed using 1% agarose gel electrophoresis [33].

### 2.4. Nucleotide Sequencing and Phylogenetic Analysis

The amplified fragment of DNA from each sample was isolated from the agarose gel through the prescribed protocol and checked on 1% agarose gel for confirmation. The isolated fragments of DNA from each sample were sent to Macrogn Inc., Seoul, Republic of South Korea, for nucleotide sequencing. After successful sequencing, the quality of the sequences was checked using Geneious software R7 (https://www.geneious.com/). The rough sequences from the start and end were deleted to ensure the quality was high [34]. Moreover, the sequencer was used for sequence editing and further analysis. The consensus sequences were used for confirmation of the correct and similar identification on the BLAST, and a Maft aligner was used to align the sequences [35]. BioEdit software (ver 7.2.0) was used to remove the roughly arranged data and MEGA 11 was used to construct a phylogenetic tree based on sequence analysis [36].

## 3. Results

**Figure 2:**
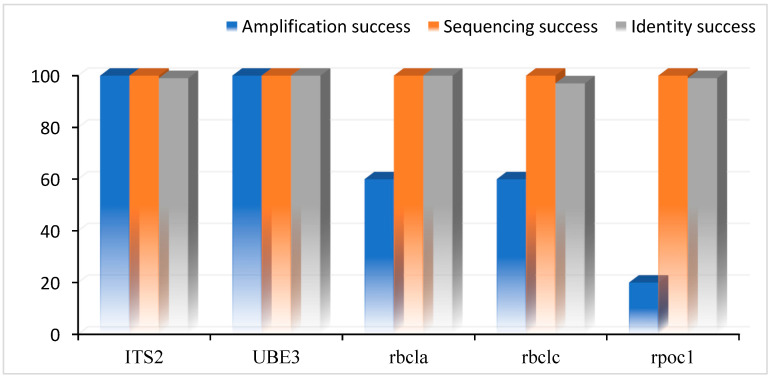
The amplification success rate, sequencing success rate, and authentication of DNA barcode region for the identification of walnut genotypes.

In the current research work, the selected walnut genotypes were evaluated using angiosperm-specific DNA markers, including nuclear (ITS2 and *UBE3*) and chloroplast (*rbcLa*, *rbcLc*, and *rpo*C*1*), which were used for molecular evaluation, differentiation, recognition, and identification. Furthermore, all the above-mentioned DNA markers demonstrated a 100% amplification rate in ITS2 and *UBE3*, 60% in *rbcLa* and *rbcLc*, and 20% in *rpo*C*1*. While successful sequencing rates were recorded at 100%, high identity success rates ranged from 97 to 100% (Figure 2).

Similar to BLAST searching the query sequences, the highest similar database sequences were compared based on query cover, percent identity, and E-value (Table 3).

### 3.1. DNA Sequence Analysis and Characterizations of Barcodes

Sequence analysis was conducted to ascertain the monophyly and evolutionary lineage of local walnut genotype samples in comparison with available database sequences. The analysis used the neighbor-joining method. The bootstrap consensus tree, based on 1000 replicates, was generated to represent the phylogeny of the genotypes. Branches supported by less than 50% of bootstrap replicates were collapsed. The percentage of replicate trees in which the associated genotypes clustered together in the bootstrap test of 1000 replicates is indicated next to the branches [37]. The Tamura–Nei method was employed to compute distances, which are presented as the number of base substitutions per site [38]. The candidate barcode sequence analysis generates various characteristics in the form of length in bp, conserved domains, variable sites, singletons, and informative sites (Table 4).

### 3.2. Nucleotide Sequence Analysis and Phylogeny of Genotype-1 Based on ITS2 

**Figure 3:**
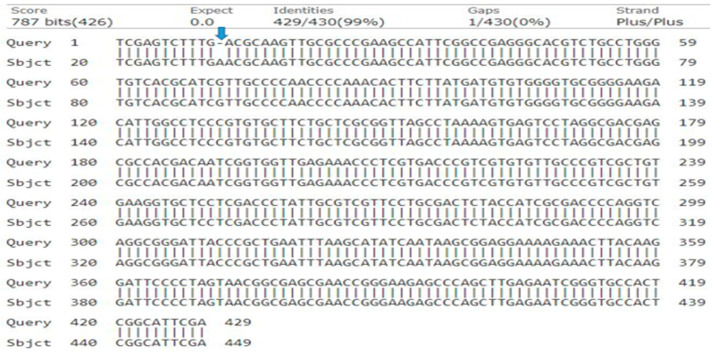
Basic local alignment of query genotype-1 ITS2 nucleotide sequence with reference database sequence, resulting in a single-nucleotide deletion (indicated by arrow) out of 430 nucleotides in position 12 of ITS2 sequence of genome.

The ITS2 region yielded a 335-base pair sequence with a query cover of 94%, 99% identity, and an E-value of 0.0. In the final dataset, characters of 335 bp, 326 conserved sites, 7 variable sites, 7 singletons, and 0 parsimony informative sites were recorded. There was a single-nucleotide missing gap recorded in blast alignment (0 = A) (Figure 3). 

**Figure 4:**
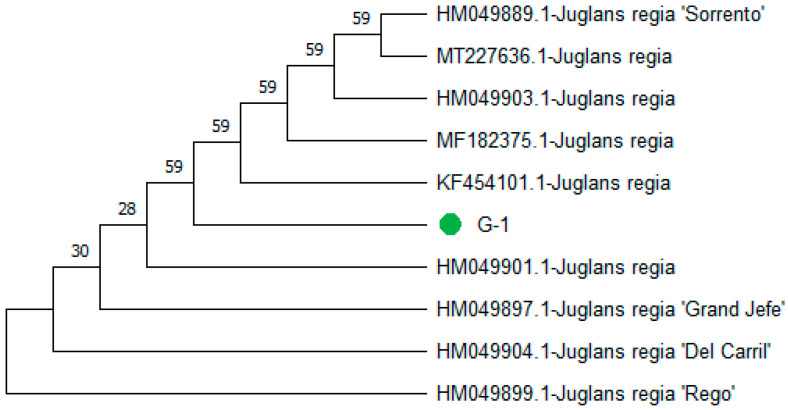
Phylogenetic tree constructed using NJ method prescribed by Saitou and Nei [39] for query nucleotide sequence of ITS2 of genotype-1 (marked with green node).

The phylogenetic analysis resulted in the formation of two clades: Clades I and II. Clade I comprised eight accessions, including (MF182375.1-*J. regia*), (HM049904.1-*J. regia* ‘Del Carril’), (HM049899.1-*J. regia* ‘Rego’), (HM049903.1-*J. regia*), (HM049891.1-*J. regia* ‘Sorrento’), and (MT227636.1-*J. regia*). The query genotype-1 sequence clustered with (KF454101.1-*J. regia*) and (MF182375.1-*J. regia*) with a bootstrap value of 59. MF182375.1-*J. regia*) is previously reported from China. Clade II included (HM049897.1-*J. regia* ‘Grand Jefe’) and (HM049901.1-*J. regia*). All sequences were retrieved from GenBank (Figure 4).

### 3.3. Nucleotide Sequence Analysis and Phylogeny of Genotype-2 Based on UBE3

**Figure 5:**
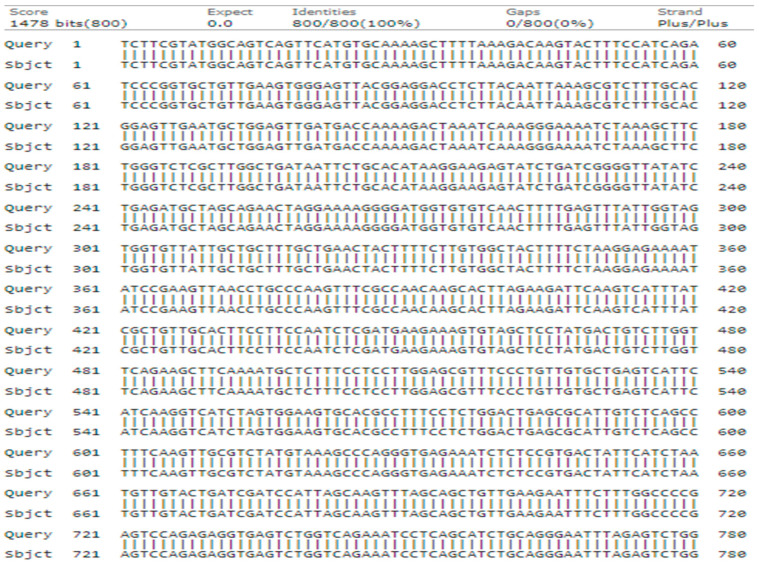
Alignment of query genotype-2 nucleotide sequence of *UBE3* with the database reference sequence.

The *UBE3* primer produced a 768-base pair sequence with a 92% query cover, 100% identity, and an E-value of 0.0. A neighbor-joining tree was constructed based on a total dataset of 768 bp, which comprised 465 conserved sites, 303 variable sites variable sites, 288 parsimony informative sites, and 15 singleton sites (Figure 5). 

**Figure 6:**
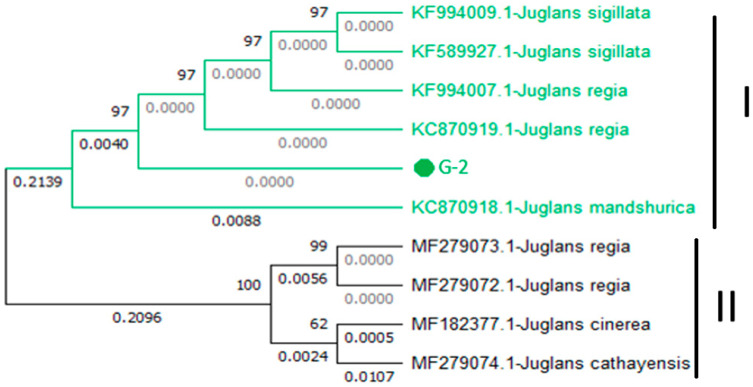
Phylogenetic tree constructed using NJ method Saitou and Nei [39] for query sequence of *UBE3* for genotype-2 (node is marked with green color).

The phylogenetic analysis revealed the formation of two major clades: Clades I and II. In Clade I, query genotype-2 shares a clade with (KF994007.1-*J. regia*), (KC870919.1-*J. regia*), (KF589927.1-*Juglans sigillata* Dode), (KF994009.1-*J. sigillata*), and (KC870918.1-*J. mandshurica)*, with a bootstrap value of 97. Both the (KF994007.1-*J. regia*) and (KC870919.1-*J. regia*) were previously reported in China in 2013. Clade II comprised four accessions, including (MF279073.1-Juglans regia), (MF279072.1-*J. regia*), (MF182377.1-*J. cinerea*), and (MF279074.1-*J. cathayensis*) (Figure 6).

### 3.4. Nucleotide Sequence Analysis and Phylogeny of Genotype-3 Based on rbcLa

**Figure 7:**
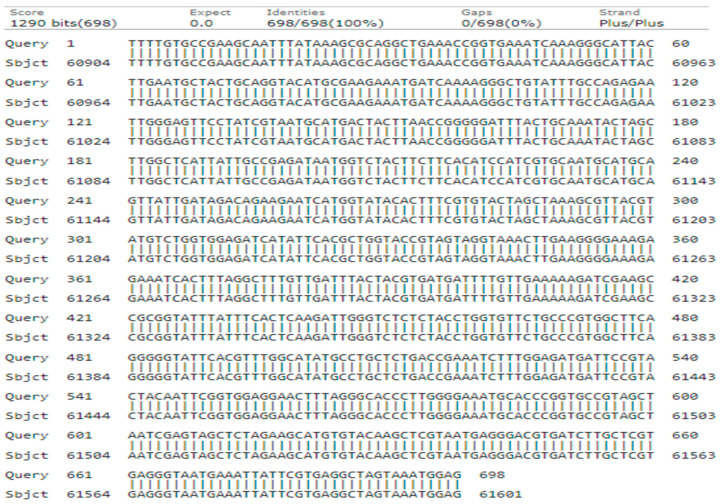
Alignment of query genotype-3 nucleotide sequence of *rbcLa* with the reference sequences collected database.

During the phylogenetic analysis, the *rbcLa* primer yielded a 698-base pair sequence with a 99% query cover, 100% identity, and an E-value of 0.0. A neighbor-joining tree was constructed based on the total dataset characters of 698 bp, which included 698 conserved sites; however, no variable parsimony informative or singleton sites were observed (Figure 7).

**Figure 8:**
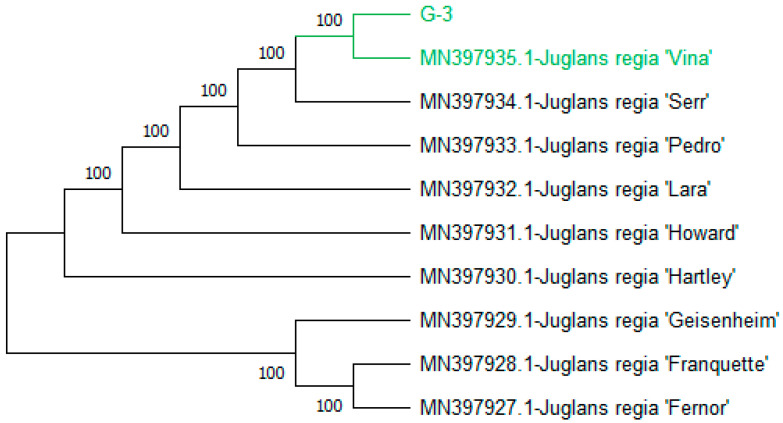
Phylogenetic tree constructed using NJ method Saitou and Nei [39] for query nucleotide sequence of *rbcLa* for genotype-3. The genotypes in green colors indicates classes occurrence.

The analysis resulted in the formation of two major clades: Clades I and II. Within clade I, query genotype-3 clustered together with a bootstrap value of 100 alongside accessions (MN397935.1-*J. regia* ‘Vina’), (MN397934.1-*J. regia* ‘Serr’), (MN397933.1-*J. regia* ‘Pedro’), (MN397932.1-*J. regia* ‘Lara’), (MN397931.1-*J. regia* ‘Howard’), and (MN397930.1-*J. regia* ‘Hartley’). (MN397935.1-*J. regia* ‘Vina’) was previously reported in Switzerland and the sequence was submitted in Ncbi in Germany in 2019. Conversely, Clade II comprised (MN397927.1-*J. regia* ‘Fernor’), (MN397929.1-*J. regia* ‘Geisenheim’), and (MN397928.1-*J. regia* ‘Franquette’) (Figure 8).

### 3.5. Nucleotide Sequence Analysis and Phylogeny of Genotype-4 Based on rbcLc

**Figure 9:**
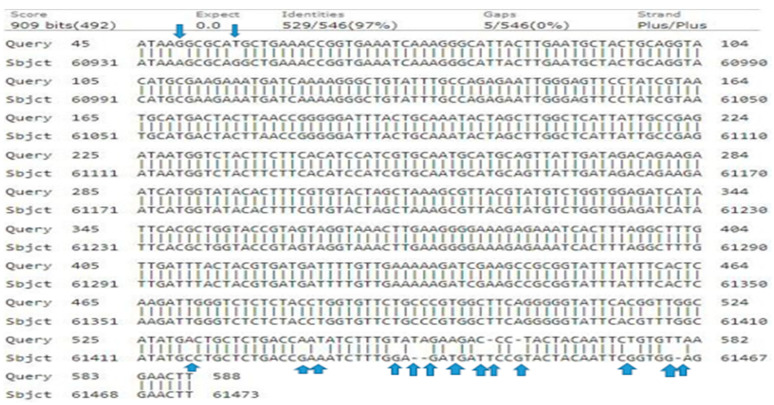
Alignment of query genotype-4 nucleotide sequence of *rbcLc* with the reference database sequences. The variations in the nucleotide’s sequences are marked by arrows.

In the phylogenetic analysis, the *rbcLc* primer produced a 587-base pair sequence with a 38% query cover, 97% identity, and an E-value of 0.0. A neighbor-joining tree was then constructed based on a total dataset of 587 bp, which consisted of 538 conserved sites, 5 variable sites, 5 singleton sites, 5 missing gap (deletion) sites (T = 0, A = 0, T = 0, T = G, T = 0), and 9 nucleotide replacement (insertion) sites (G = A, T = G, A = C, A = G, T = A, C = T, T = G, and =G) (Figure 9).

**Figure 10:**
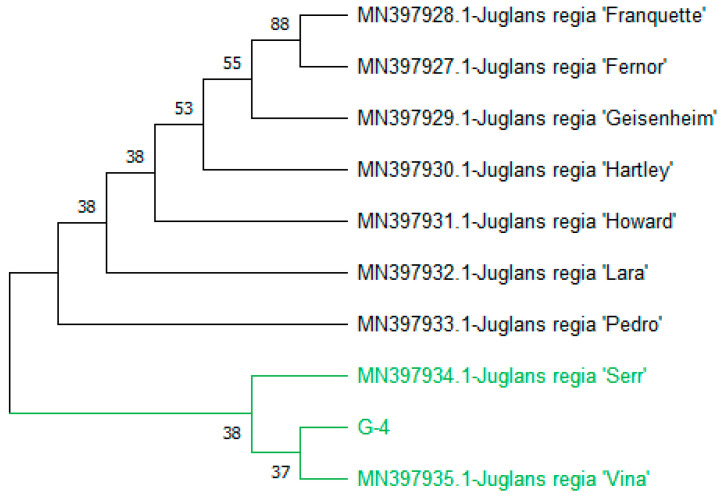
Phylogenetic tree constructed using NJ method Saitou and Nei [39] for query sequence of *rbcLc* genotype-4. The Genotypes in the green colors alongside of G-4 are the closest and previously reported.

The phylogeny revealed that the total accessions clustered into two distinct clades: Clades I and II. Clade I comprised seven accessions, namely (MN397928.1-*J. regia* ‘Franquette’), (MN397927.1-*J. regia* ‘Fernor’), (MN397929.1-*J. regia* ‘Geisenheim’), (MN397930.1-*J. regia* ‘Hartley’), (MN397931.1-*J. regia* ‘Howard’), (MN397932.1-*J. regia* ‘Lara’), and (MN397933.1-*J. regia* ‘Pedro’). In Clade II, query genotype-4 clustered with (MN397935.1-*J. regia* ‘Vina’) and (MN397934.1-*J. regia* ‘Serr’) with bootstrap values of 37 and 38. Both the (MN397935.1-*J. regia* ‘Vina’) and (MN397934.1-*J. regia* ‘Serr’) were previously reported in Switzerland and sequences were submitted in Germany in 2017 and 2019 (Figure 10).

### 3.6. Nucleotide Sequence Analysis and Phylogeny of Genotype-5 Based onrpoC1

**Figure 11:**
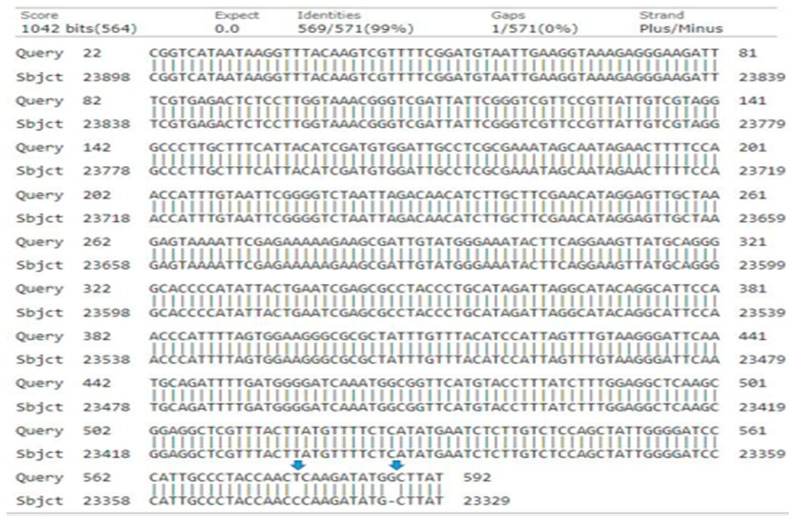
Alignment of query genotype-5 nucleotide sequence of *rpoC1* with the reference sequence collected from database. The variables nucleotides are marked with arrows.

In the phylogenetic analysis, the *rpoC1* primer yielded a 646-base pair sequence with a 58% query cover, 99% identity, and an E-value of 0.0. A neighbor-joining tree was then constructed based on a total dataset of 646 bp, which included 331 conserved sites, 239 variable sites, 239 singleton sites, 1 missing gap deletion (G = 0), and 1 nucleotide replacement (insertion) of T = C (Figure 11).

**Figure 12:**
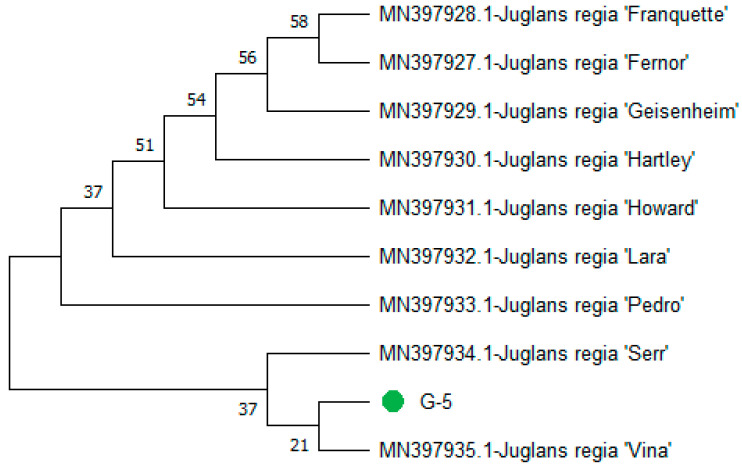
Phylogenetic tree constructed using NJ method Saitou and Nei [39] for query sequence of *rpoC1* genotype-5 (green node).

The resulting phylogeny grouped the total accessions into two distinct clades: Clades I and II. Clades I consisted of seven accessions, namely (MN397928.1-*J. regia* ‘Franquette’), (MN397927.1-*J. regia* ‘Fernor’), (MN397929.1-*J. regia* ‘Geisenheim’), (MN397930.1-*J. regia* ‘Hartley’), (MN397931.1-*J. regia* ‘Howard’), (MN397932.1-*J. regia* ‘Lara’), and (MN397933.1-*J. regia* ‘Pedro’). In Clade II, query genotype-5 clustered with bootstrap values of 28 (MN397935.1-*J. regia* ‘Vina’) and 41 (MN397934.1-*J. regia* ‘Serr’) (Figure 12).

### 3.7. DNA Barcode of Selected Genotypes

**Figure 13:**
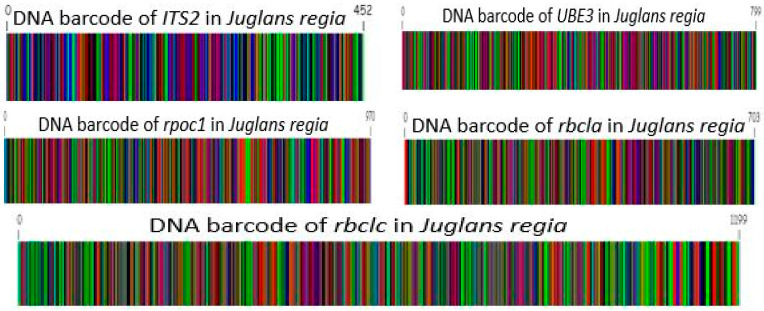
DNA barcode of selected markers selected walnut genotypes.

The barcode from the nuclear (ITS2 and *UBE3*) and chloroplast (*rbcLa*, *rbcLc*, and *rpoC1)* marker sequence was generated by DNA barcode generator (Bio-Rad) software (v 1.3) for their exact identification (Figure 13).

## 4. Discussion

The present research work was designed to screen out suitable DNA barcode regions for available cryptic walnut genotypes and check the phylogenetic relationship with reference database sequences. Walnut genotypes were collected from the different localities of the Hazara region of Pakistan and evaluated through different DNA markers. A combination of different molecular markers provides comprehensive insights into walnut diversity [40]. In the current study, various types of nuclear (ITS2 and *UBE3*) and chloroplast (*rbcLa*, *rbcLc,* and *ropC1*) were used to confirm the amplification sequencing and identity success rate in the selected cryptic walnut genotypes. Similarly, various types of DNA markers have been used in previous studies on *Juglans* species by Yang et al. [41]. 

In the current study’s results, primers reveal notable performances in the successful amplification rate, sequencing rate, and rate of similarity among the tested DNA barcoding markers in local walnut genotypes. ITS2, *UBE3*, *rbcLa*, *rbcLc*, and *rpoC1* markers performed well across all metrics, with maximum amplification, sequencing, and identity success rates. Similar to the previous study, high amplification and sequencing success rates of selected DNA markers were reported by Chen et al. [42]. The current study’s results regarding the identification success rate of 97–100% (>90% identity, >90% query cover, and E value of 0.0) align with the results underscoring the reliability of ITS2 and *rbcL* in genus *Juglans* L. by Han et al. [43], *Juglans nigra* L. by Pollegioni et al. [40], and *UBE3* by Suo et al. [26] in *J. regia*. L cultivars, *rbcL*, and *rpo*C*1* (Aradhya et al. [44]) in *Juglans* species identification. Our analysis revealed a significant number of conserved sites (326, 465, 698, 538, and 331 in ITS2, *UBE3*, *rbcLa*, *rbcLc*, and *rpoC1*) and 303 and 239 variable sites in *UBE3* and *rpoC1* markers. Comparable to the findings of Stevens et al. [45] who also documented genetic variations in *Juglans* species, resolved phylogenetic relationships within *Juglans,* and identified specific regions, conserved sites are useful for species identification and evolutionary studies of *Juglans* species. 

The nucleotide gaps (deletions) 4 (T = 0) 1 (A = 0) and nucleotide replacements (substitutions) 3 (T = G) 2 (G = A) 1 (A = C) 1 (T = A) 1 (C = T) were identified particularly in *rbcLc* and 1 (G = 0) 1 (T = C) and A = 0 in *rpo*C*1*. The current study is like those reported, highlighting the genetic complexity within walnut species [46]. This study also contributes to genetic diversity by introducing new variations that can be acted upon by evolutionary forces [6]. Variations such as nucleotide substitutions and deletions can lead to a reproductive barrier, causing them to be reproductively isolated from other populations and leading to speciation [47]. Non-synonymous substitutions and significant deletions can lead to an adaptation that allows different populations to exploit different ecological niches. Over time, this ecological differentiation can contribute to the formation of new species [48]. Genetic markers like ITS2, *UBE3*, *rbcL*, and *ropC1* are often used in phylogenetic studies to understand the evolutionary relationships between species. Variations in these markers can help trace lineage divergence and speciation events [49]. The phylogenetic tree constructed using the NJ method showed robust clustering of query genotypes with *J. regia* and cultivars of *J. regia* with high bootstrap scores. The ITS2 sequence-based phylogenetic tree results in the formation of two major clades, Clades I and II. In Clade I, the query genotype sequence clustered with (KF454101.1-*J. regia*) with a bootstrap value of 90%. (KF454101.1-*J. regia*) was previously reported or submitted in northern China by Wang et al. [50]. In the phylogeny, the query genotypes clustered with (KF994007.1-*J. regia*) and (KC870919.1-*J. regia*), with a bootstrap value of 97. Both the sequences of species (KF994007.1-*J. regia*) and (KC870919.1-*J. regia*) have been previously reported in China [26]. On the other hand, phylogeny, query genotype-3 clustered with (MN397935.1-*J. regia* ‘Vina’), (MN397934.1-*J. regia* ‘Serr’), (MN397933.1-*J. regia* ‘Pedro’), (MN397932.1-*J. regia* ‘Lara’), (MN397931.1-*J. regia* ‘Howard’), and (MN397930.1-*J. regia* ‘Hartley’) with a bootstrap value of 100. All these *J. regia* cultivars were previously reported by Teske et al. [51] in Germany. With phylogeny based on *rbcLc* and *rpoC1*, query genotype-4 and genotype-5 clustered with (MN397935.1-*J.* regia ‘Vina’) and (MN397934.1-*J. regia* ‘Serr’). Both (MN397934.1-*J. regia* ‘Serr’) and (MN397935.1-*J. regia* ‘Vina’) were previously reported by Teske et al. [51] in Germany. These results supported the monophyly of *Juglans* and provided insights into the evolutionary relationships within the genus. The current research is also supported by several studies using different kinds and combinations of DNA markers used for amplification, sequencing, and walnut species identification.

Similarly, 113 walnut genotypes from different regions of China were analyzed for genetic diversity and population structure analysis using molecular markers [50]. Aradhya et al. [44] examined 77 walnut cultivars for genetic differentiation and conservation using DNA markers. Similarly, in a previous study, ITS2 was used along with chloroplast DNA to analyze phylogenetic relationships within *Juglans*, helping to identify species and cultivars [52]. On the other hand, Yildiz et al. [53] analyzed the genetic diversity and identified the *J. regia* and its related species through various markers. Similarly, Chen et al. [42] identified the phylogenetic relationship and fingerprint elite cultivars through molecular markers in *Juglans.* Nuclear ITS and chloroplast DNA sequences are reliable for phylogenetic studies in *Juglans* [27]. Aradhya et al. [44] applied ITS and chloroplast DNA sequences to construct a molecular phylogeny of *Juglans*, offering a biogeographic perspective on the genus. Similarly, ITS2 markers were used for the phylogenetic analysis and diversification of the order Fagales [54], the molecular characterization, genetic diversity, and phylogenetic relationships of walnut genotypes [55], the genetic diversity and evolutionary relationships within the walnut germplasm in Azerbaijan [56], and the population structure and genetic diversity of walnuts in Kazakhstan. Genome sequences provide insight into the genetic diversity and potential markers for cultivar identification in walnuts [57]. These findings emphasize the robustness and reliability of effective markers for DNA barcoding, highlighting their potential for accurate species identification and genetic diversity analysis within walnut populations. Similarly, the current findings confirm the efficacy of markers in the identification of the taxon at the species level. However, the literature on *UBE3*, *rbcLa*, *rbcLc*, and *rpoC1* as DNA barcoding markers for walnut genotypes is limited; studies on other plant species have highlighted their efficacy in accurate species identification and phylogenetic inference [58].

## 5. Conclusions

The DNA barcode regions we used were successfully amplified using PCR, successfully sequenced, managed, and analyzed through different computational software and tools. The differentiation and recognition of query genotype sequences were carried out through analysis and comparisons with the database sequences of the genus *Juglans*. The overall primer efficacy surpassed 90%. The nuclear markers applied, i.e., ITS2 and *UBE3,* and chloroplast markers, i.e., *rbcLa*, *rbcLc,* and *rpoC1*, performed best for the evaluation of walnut genotypes and are recommended for the evaluation of other *Juglans* genotypes across the world. The phylogenetic analysis revealed that all the query genotypes belong to the genus *Juglans* and *regia (J. regia)* (KF454101.1-*J. regia*) and (KC870919.1-*J. regia)* and cultivars of the *Juglan regia* such as (MN397935.1-*J. regia* ‘Vina’), (MN397934.1-*J. regia* ‘Serr’), (MN397933.1-*J. regia* ‘Pedro’), (MN397932.1-*J. regia* ‘Lara’), (MN397931.1-*J. regia* ‘Howard’), and (MN397930.1-*J. regia* ‘Hartley’). The current findings provide baseline information for the next steps in research with respect to the genus *Juglans* genotypes and their recognition, diversity, occurrence, habit, habitat, and use for cultivation, grafting, and breeding programs. 

## Figures and Tables

**Table 1:** Collection sites of genotypes studied.

Genotypes	Collection Sites	Latitude (N)	Longitude (E)
Genotype-1	Shingli bala Battagram	34°40′42″	72°59′05″
Genotype-2	Sanger valley Kaghan	34°34′55″	73°22′43″
Genotype-3	Jared valley Kaghan	34°40′34″	73°33′24″
Genotype-4	Rashang valley Allai	34°49′10″	73°7′30″
Genotype-5	Shatial upper Kohistan	35°31′37″	73°32′36″

**Table 2:** DNA barcode regions and their nucleotide sequences used.

Barcode Region	Primers	Sequence 5′–3′	References
ITS2	F	5′ATGCGATACTTGGTGTGAAT3′	[25]
*UBE3*	F	5′TCGCCTCCAAGTTCAGTG3′	[26]
*rbcLa*	F	5′ATGTCACCACAAACAGAGACTAAAGC3′	[27]
*rbcLc*	F	5′TGAAAACGTGAATTCCCAACCGTTTATGCG3′	[28]
*rpoC1*	F	5′AATCTATGCAGGGTAGGCGC3′	[29]

**Table 3:** The sequence lengths, query covers, E-values, and percent identity of studied DNA barcode regions.

Primers	Sequence (bp)	Query Cover	E-Value	% Identity
ITS2	335	94	0	99.77
*UBE3*	768	92	0	100
*rbcLa*	698	99	0	100
*rbcLc*	587	38	0	96.89
*rpoC1*	646	58	0	99.65

**Table 4:** Molecular characterizations of candidate’s DNA barcode regions in selected genotypes.

Barcode Region	Total Characters	Conserved Sites	Variable Site	Parsimony Info	Singleton
ITS2	335	326	7	0	7
*UBE3*	768	465	303	288	15
*rbcLa*	698	698	0	0	0
*rbcLc*	587	538	5	5	5
*rpoC1*	646	331	239	0	239

## Data Availability

The original contributions presented in the study are included in the article, further inquiries can be directed to the corresponding authors.
